# Extensive Morphea Following Adjuvant Radiotherapy for Breast Carcinoma—Case Report

**DOI:** 10.3390/curroncol32010050

**Published:** 2025-01-18

**Authors:** Alexandru Panaitescu, Hannah Nguyen, Laurence Masson-Côté, Carolina Lucena Fernandes

**Affiliations:** 1 Radiation Oncology Service, Department of Nuclear Medicine and Radiation Oncology, Faculty of Medicine and Health Sciences, Université de Sherbrooke, Sherbrooke, QC J1H 5N4, Canada; alexandru.panaitescu@usherbrooke.ca (A.P.); laurence.masson-cote@usherbrooke.ca (L.M.-C.); 2 Dermatology Service, Department of Medicine, Faculty of Medicine and Health Sciences, Université de Sherbrooke, Sherbrooke, QC J1H 5N4, Canada; hannah.nguyen@usherbrooke.ca

**Keywords:** radiation-induced morphea, lichen sclerosis, Koebner phenomenon, radiation therapy, breast cancer, oncodermatology

## Abstract

Radiation-induced morphea (RIM) is a rare complication following radiotherapy (RT) for breast cancer treatment. Its distribution is usually confined to the breast having received radiotherapy. A generalized form of RIM also exists, defined as lesions extending beyond the radiotherapy site, but data on the subject are scarce in the literature. This complication remains difficult to treat, due partly to the variable extent of disease and to individual clinical response rates to the wide array of available therapies, such as topical therapy (i.e., topical tacrolimus or topical corticosteroids), phototherapy, and systemic therapy (i.e., systemic immunosuppressants). We present a case of extensive morphea post RT for breast cancer with 2 years of favorable evolution under systemic therapy.

## 1. Introduction

Certain skin complications have been widely described in the literature following the use of radiotherapy (RT) for breast cancer, such as moist desquamation, erythema or radiodermatitis, and post-irradiation fibrosis [1]. A rare but possible complication following adjuvant RT is radiation-induced morphea (RIM) [2], a localized inflammatory cutaneous sclerosis of the dermis that may extend to subcutaneous tissues. It is often limited to the RT treatment site, appearing months to years after the completion of RT [3]. Because of its rare occurrence, radiation-induced morphea can often be mistaken for other diagnoses, including cancer relapse, radiation-induced fibrosis and infection [4]. While this disease is more commonly found in patients receiving RT for breast carcinoma, it has also been described to a lesser extent in a variety of other cases of cancer receiving radiation treatment, such as lymphoma and gastric cancer [5]. Although localized RIM manifesting as an inflammatory process limited to the radiation site is well documented, few cases of generalized RIM have been reported. We present here a case of generalized morphea following RT for breast carcinoma and a review of the associated existing literature. 

## 2. Detailed Case Description

A 77-year-old woman presented with a 5-month history of bilateral breast deformities, with her left side being more affected than the right. Previous medical history was significant for hypothyroidism treated with Synthroid and for left breast carcinoma diagnosed 4 years prior, in 2018. Staging was classified as pT1cN0M0, with positive estrogen receptors, negative progesterone receptors, and positive HER2 receptors. The patient underwent partial mastectomy with sentinel lymph node biopsy and only one cycle of adjuvant chemotherapy (docetaxel, cyclophosphamide, and trastuzumab). It was discontinued early because of significant side effects. She then received standard adjuvant 3-D conformal external beam radiation therapy (3D-CRT) to the whole left breast (WBI) with a dose of 40 Gy delivered in 15 fractions, followed by a boost dose of 10 Gy in 4 fractions focused on the tumour bed, completed in April 2018. Acute RT side effects consisted of grade 2 radiation dermatitis with moist desquamation in the inframammary fold, which resolved within a few weeks with standard routine care. She was then prescribed adjuvant anastrozole for 5 years and had no evidence of cancer recurrence since. 

**Figure 1:**
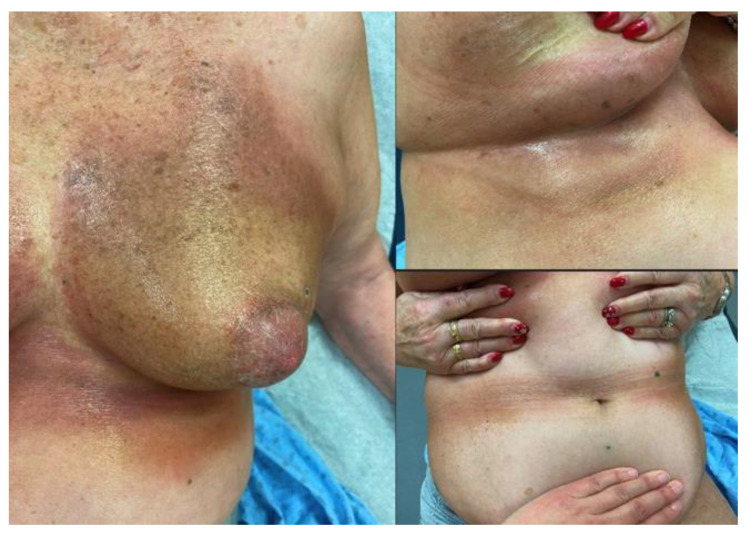
Erythematous-violaceous plaques, with cardboard-like skin changes causing deformities of the left breast. Lesions extend to bilateral inframammary areas and abdominal folds with Koebner phenomenon on frictional sites related to the garments’ elastic bands.

Four years later, in July 2022, she noticed erythematous and violaceous skin changes on her left breast, which evolved into cardboard-like texture with pruritus and severe bilateral breast deformities in the following 3 months. Skin changes started in the left inframammary region and then extended to the right side, followed by the abdominal folds and along the spine (Figure 1).

Koebner phenomenon was noted on frictional sites. The patient presented no symptoms of connective tissue disease and no palpable lymph nodes. A breast ultrasound revealed diffuse thickening of the skin up to the adipose tissue, without any underlying suspicious breast lesion. A skin biopsy revealed dermal fibrosclerosis and chronic inflammatory remodeling, with epidermal atrophy as well as endothelial and fibroblastic remodeling. A mammogram, breast resonance imaging and ultrasound, physical examination, and skin investigation showed no evidence of breast cancer relapse.

Dermatologic diagnosis suggested a generalized morphea with progression beyond the RT treatment site. The patient was initially treated with prednisone, high potent topical corticosteroids and topical vitamin D, as well as regular phototherapy sessions. Since skin lesions had only partially improved after a year of treatment, and breast deformities were still present, systemic mycophenolate mofetil 1 g PO BID was then added. As of April 2024, this immunosuppressive agent is well tolerated by the patient, with a significant control of skin disease and symptom relief. The patient’s characteristics and treatment trajectory are summarized in Table 1 found below.

## 3. Discussion

Skin changes, such as desquamation, erythema and fibrosis, are common side effects of RT for breast cancer. RIM, however, is a rarer complication that may occur, with its incidence corresponding to around 1 in 378 [4]. RIM usually starts shortly after RT or in the years following it. This disease typically begins with erythematous plaques present at the treatment site that progress to sclerotic, indurated plaques. It can involve deep dermis and subcutaneous fat. Joint movement limitation and muscle atrophy may be seen, depending on the affected region [1]. While this condition is not life-threatening, it can greatly impact the quality of life of individuals who are affected by this disease, with pain, fatigue and joint restriction being the most common symptoms reported by patients.

The generalized presentation found in this case remains an even more rare presentation of RIM, since there are only a few reports documenting this phenomenon [4,5]. In their review of the available literature completed in 2017, Gonzalez-Ericsson et al. described only 24 cases extending beyond the irradiated field appearing since 1989 [6]. This extensive form of RIM could appear as indurated, hyperpigmented, discolored, and painful lesions. Affected areas included the opposite breast [7], the trunk [5,8], the axilla and the arm [9] of the same side, as well as the lower limbs [5]. One case described fatigue and generalized arthralgias [8]. Such a low reported number suggests that this condition is rare. However, it may be possible that RIM is either undiagnosed or misdiagnosed. Therefore, it is important to quickly recognize this rare manifestation to initiate proper treatments. 

Although the exact pathophysiological mechanism behind the development of RIM remains unclear, some previous studies in the existing literature lay out the hypothesis that the formation of new antigens post-radiotherapy can stimulate key components of the immune response [10]. As a direct result of radiation therapy, different alterations are made on cellular proteins, leading to the formation of these new antigens [11]. These neoantigens are then recognized by key components of the immune system, thus triggering a T-cell response that induces more fibroblast activation through the release of TGF-α. Through a repetitive feedback loop, collagen production is maintained, leading to the development of RIM [10]. 

Constant rubbing between the skin and the clothes may elicit lesions such as the ones seen on the patient’s abdominal folds. This dermatological response is called Koebner phenomenon and may be observed in different skin conditions, such as psoriasis, lichen planus as well as morphea [12]. 

Specific risk factors have been linked to the development of RIM in the female population treated for breast cancer including patient’s smoking history, obesity and larger breast size. An association with inflammatory diseases, such as Hashimoto’s thyroiditis, connective tissue disease, and rheumatoid arthritis have also been reported [13]. Use of a boost dose, site of RT, fractioning scheme of RT, and age have not been shown to be related to the development of RIM [9,14]. 

In their 2015 review of the literature, Spalek et al. retrieved 61 cases of RIM, with 52 of these cases being reported in females receiving breast radiotherapy [14]. RIM was also reported in other cancer types, such as gastric carcinoma, lymphoma, endometrial cancers, head and neck cancers and cervical cancers, although to a much lesser extent [14]. A proposed hypothesis for the strong association found between RIM and breast radiotherapy would be the proximity of the radiation treatment site to entry points in the skin—by both cutis and subcutaneous tissue [15]. Another possible explanation includes the difficulty in diagnosing RIM in other sites of radiotherapy that are unapparent [14]. 

Diagnosis of RIM is based on clinical findings and skin biopsy, as laboratory results are usually normal. In its early stages, skin pathology shows inflammatory changes, with perivascular lymphocytic and plasmocytic infiltrates of the dermis and subcutaneous fat. As the morphea progresses, fibrosis of the dermis and subcutaneous tissue become more prominent [16].

A wide array of therapeutical approaches can be tried for patients presenting with RIM, as clinical response varies greatly depending on each individual and on the extent of the condition. The first therapeutical line for RIM includes local or intralesional therapies, such as topical corticosteroids and topical calcineurin inhibitors [3]. For more extensive or resistant cases of RIM, topical treatment and phototherapy may not be sufficient [14]. Thus, systemic therapies can be used to achieve clinical response and control of the lesions [15,16]. Immunosuppressive therapy options include mycophenolate mofetil and methotrexate [5,14]. In the rare cases of generalized morphea following RT in breast cancer patients, the use of these systemic agents may effectively control lesions and associated symptoms [8,14]. In our case, the risks related to the introduction of an immunosuppressive therapy and its potential impact on breast cancer relapse needed to be considered together with the necessity of treating a refractory disease. After a multidisciplinary discussion, it was eventually deemed safe to undergo such treatment as long as close follow-ups were set-up. In fact, the patient had generalized refractory lesions, had been cancer-free for 4 years, and was compliant to adjuvant hormonal therapy with anastrozole. 

## 4. Conclusions

This is a rare case of RIM extending beyond the radiation treatment site in breast carcinoma. While response to treatment greatly varies in the literature regarding patients afflicted by RIM, systemic agents and immunosuppressors, such as mycophenolate mofetil, can be used. They are effective therapeutic lines to achieve clinical response in cases of generalized morphea as presented above. When presented with similar cardboard-like lesions on the breast, it remains important to eliminate a potential recurrence of breast carcinoma from our differential diagnosis.

## Figures and Tables

**Table 1:** Patient characteristics and treatment trajectory.

Characteristic	Value
Age at RIM diagnosis (years)	77
Sex	Female
Past medical history (other)	Hypothyroidism treated with Synthroid, essential tremors
Past dermatological conditions	None
Oncological condition	Invasive ductal carcinoma, left breast, grade 2, pT1cN0M0, diagnosed at 73 years in 2018
Treatments for oncological condition	Partial mastectomy with sentinel lymph node biopsy; one cycle of adjuvant chemotherapy (docetaxel + cyclophosphamide + trastuzumab); 3D-CRT WBI 40 Gy/15 fractions + tumour bed boost 10 Gy/4 fractions; adjuvant Anastrozole
Time between administration of RT and onset of RIM (years)	4
RIM progression time (months)	3
Investigation performed to confirm RIM diagnosis	Skin biopsy
Investigations performed to exclude breast carcinoma recurrence (RIM’s main differential diagnosis)	Mammogram, breast ultrasound and MRI
Treatments given for RIM management	Trial of prednisone, Clobetasol, Dovonex with regular narrow-band UVB (TL-01) phototherapy sessions. After partial improvement of lesions for over a year, addition of systemic mycophenolate mofetil 1 g PO BID
Time needed to achieve remission (months)	25
Risk of cancer recurrence associated with immunosuppression and RT	Discussed in tumour board review: low risk, close clinical and imaging surveillance recommended

## Data Availability

The data presented in the study are available in this article.
